# Supplementary figures and images for: Plxdc family members are novel receptors for the rhesus monkey rhadinovirus (RRV)
Source: PLoS Pathog. 2021 Mar 3;17(3):e1008979. doi: 10.1371/journal.ppat.1008979 (PMC7959344; doi:10.1371/journal.ppat.1008979)

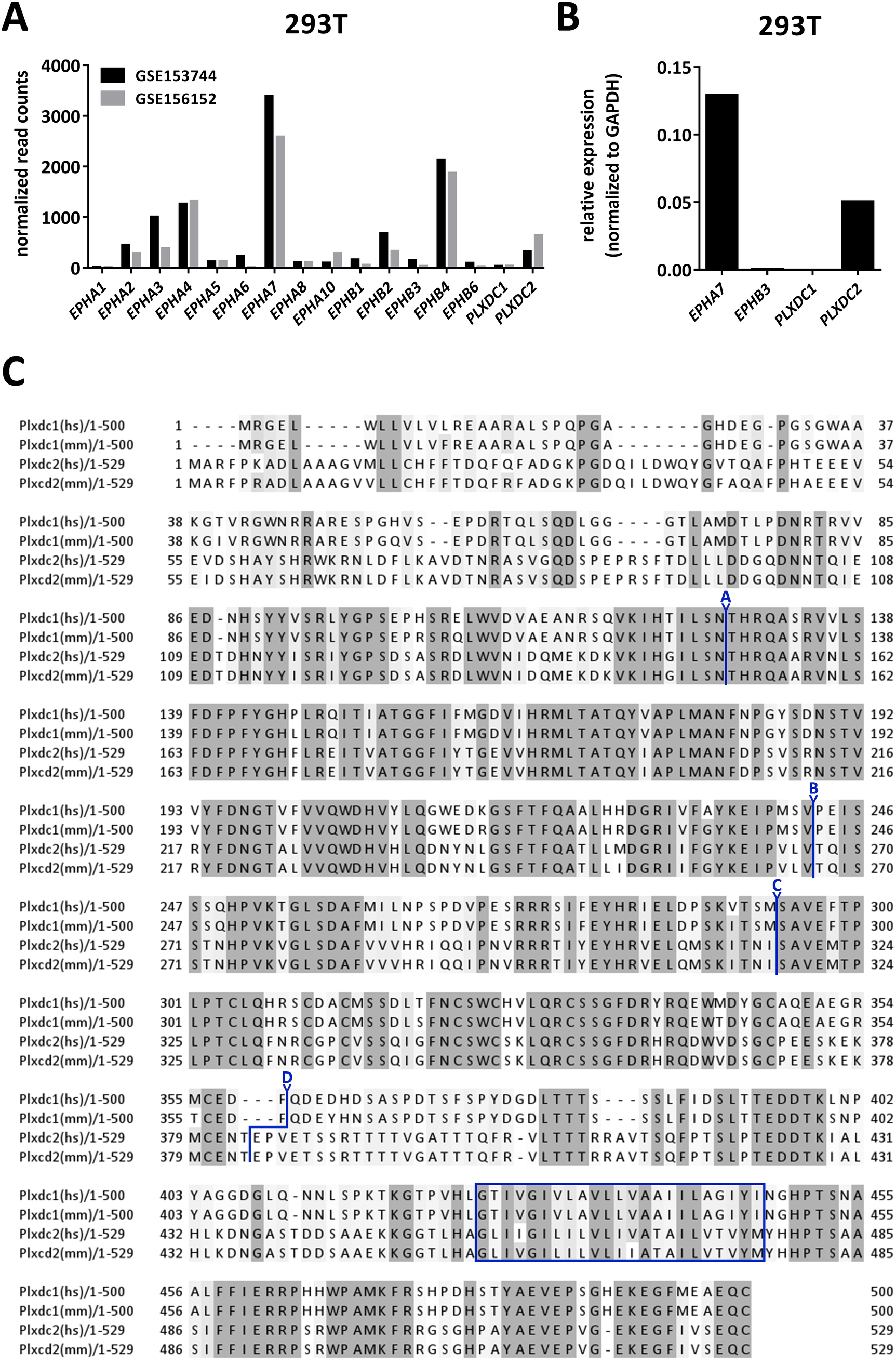

Supplement: S1 Fig — A) Normalized read counts of the 14 EPH receptor genes, PLXDC1 and PLXDC2 as found in the GEO data set series GSE153744 (HEK 293T DMSO rep1-4, GSM4652564, GSM4652566, GSM4652568, GSM4652569) and GSE156152 (Mock-1-3, GSM4725672, GSM4725673, GSM4725674). B) Expression of selected EPH genes, PLXDC1 and PLXDC2 analyzed by qPCR in 293T cells. C) Alignment of human (hs) and rhesus macaque (mm) Plxdc1 and Plxdc2. Junctions of domains (blue letters and lines) and the putative transmembrane domain (blue box) as described by Cheng et al. [31] are indicated. (TIF) [file ppat.1008979.s001.tif]

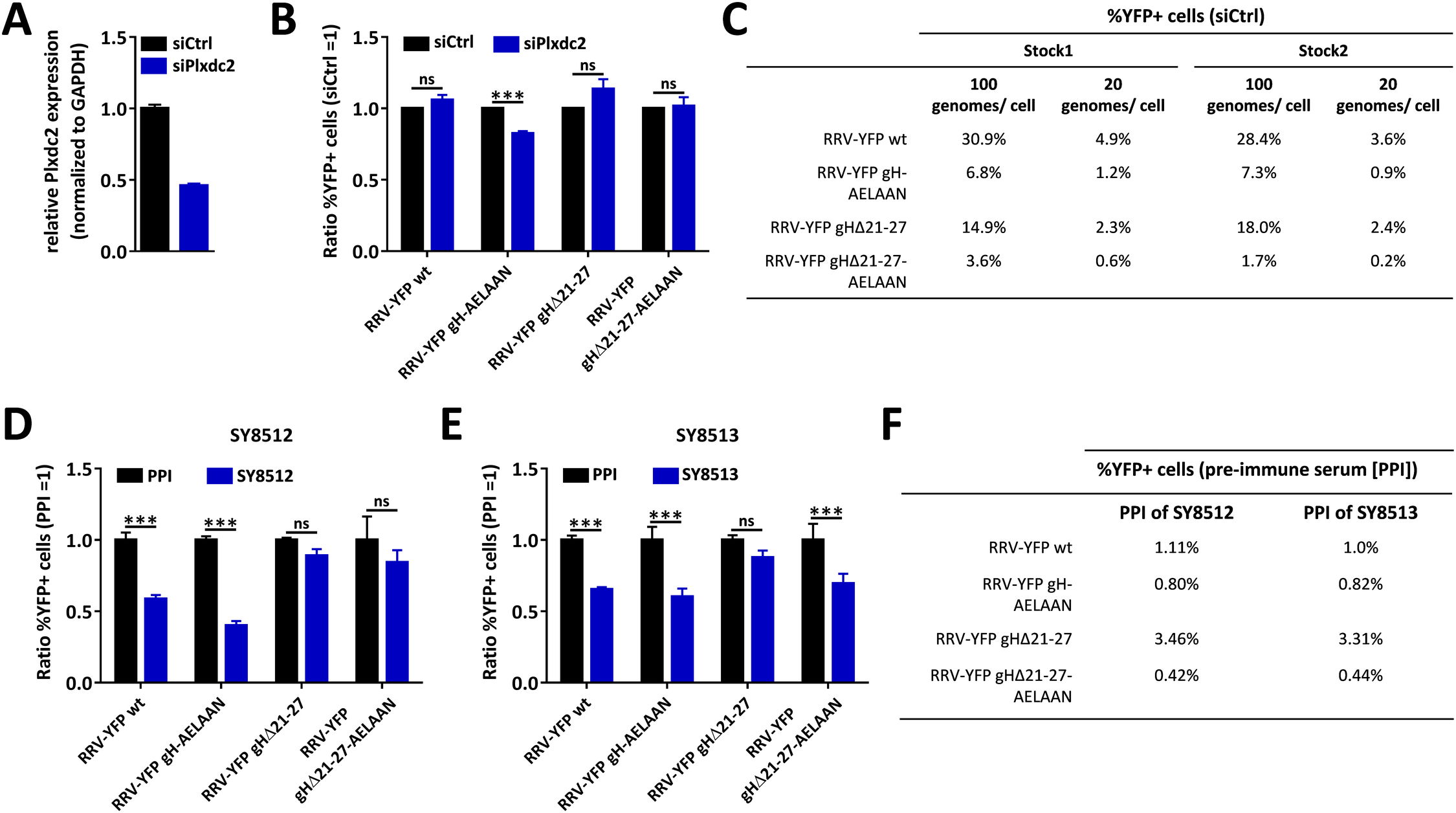

Supplement: S2 Fig — A-C) 293T cells were treated with siRNA against PLXDC2 (siPlxdc2) or control siRNA (siCtrl) for 72h. PLXDC2 expression was assessed using qPCR (A). Values were normalized to GAPDH expression and are shown relative to siCtrl. The cells were infected with RRV-YFP wt or mutants as indicated (B). YFP expression as indicator of infection was measured by flow cytometry. Infection was normalized to infection of siCtrl treated 293T cells. The mean relative infection of two sets of RRV stocks in two dilutions is shown. Absolute infection rates of 293T siCtrl cells for all stocks and dilutions are given as %YFP+ cells in C. D-F) 293T cells were pre-incubated with rabbit serum raised against recombinant Plxdc2 (two animals: SY8512, SY8513) or pre-immune serum (PPI) of the same animals for 30min prior to infection with RRV-YFP wt or the indicated mutants. YFP expression as indicator of infection was measured by flow cytometry (triplicates, error bars represent SD). Absolute infection rates of 293T cells treated with pre-immune serum are given as %YFP+ cells in F. (TIF) [file ppat.1008979.s002.tif]

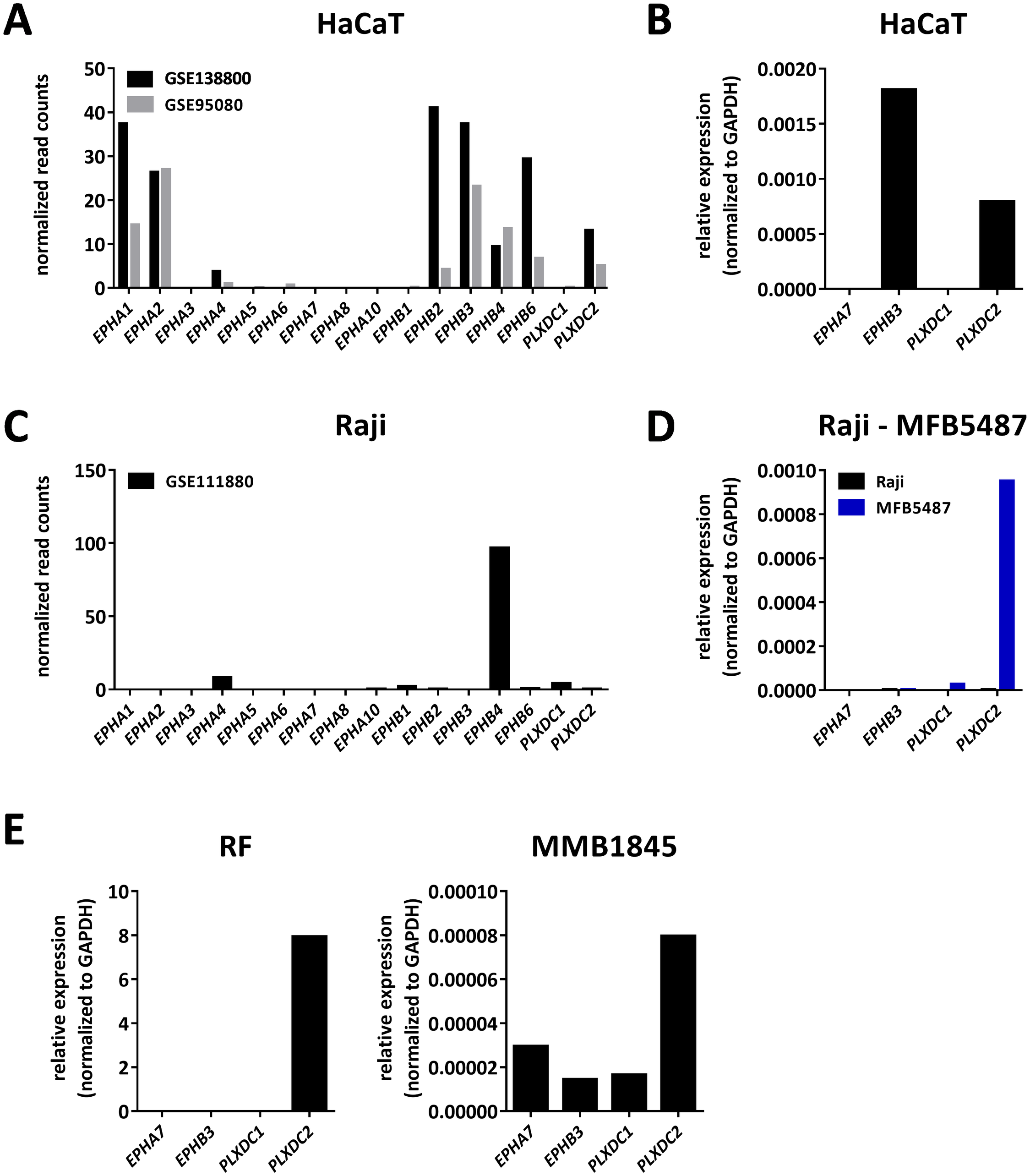

Supplement: S3 Fig — A) Normalized read counts of the 14 EPH receptor genes, PLXDC1 and PLXDC2 as found in the GEO data set series GSE138800 (C1-C3, GSM4119632, GSM4119633, GSM4119634) and GSE95080 (Uninfected HaCaT, GSM2495796). B) Expression of selected EPH genes, PLXDC1 and PLXDC2 analyzed by qPCR in HaCaT cells. C) Normalized read counts of the 14 EPH receptor genes, PLXDC1 and PLXDC2 as found in the GEO data set series GSE111880 (Raji total RNA (replicate1-4), GSM3043273, GSM3043274, GSM3043275, GSM3043276). D) Expression of selected EPH genes, PLXDC1 and PLXDC2 analyzed by qPCR in Raji and MFB5487 cells. E) Expression of selected EPH genes, PLXDC1 and PLXDC2 analyzed by qPCR in RF and MMB1845 cells. (TIF) [file ppat.1008979.s003.tif]

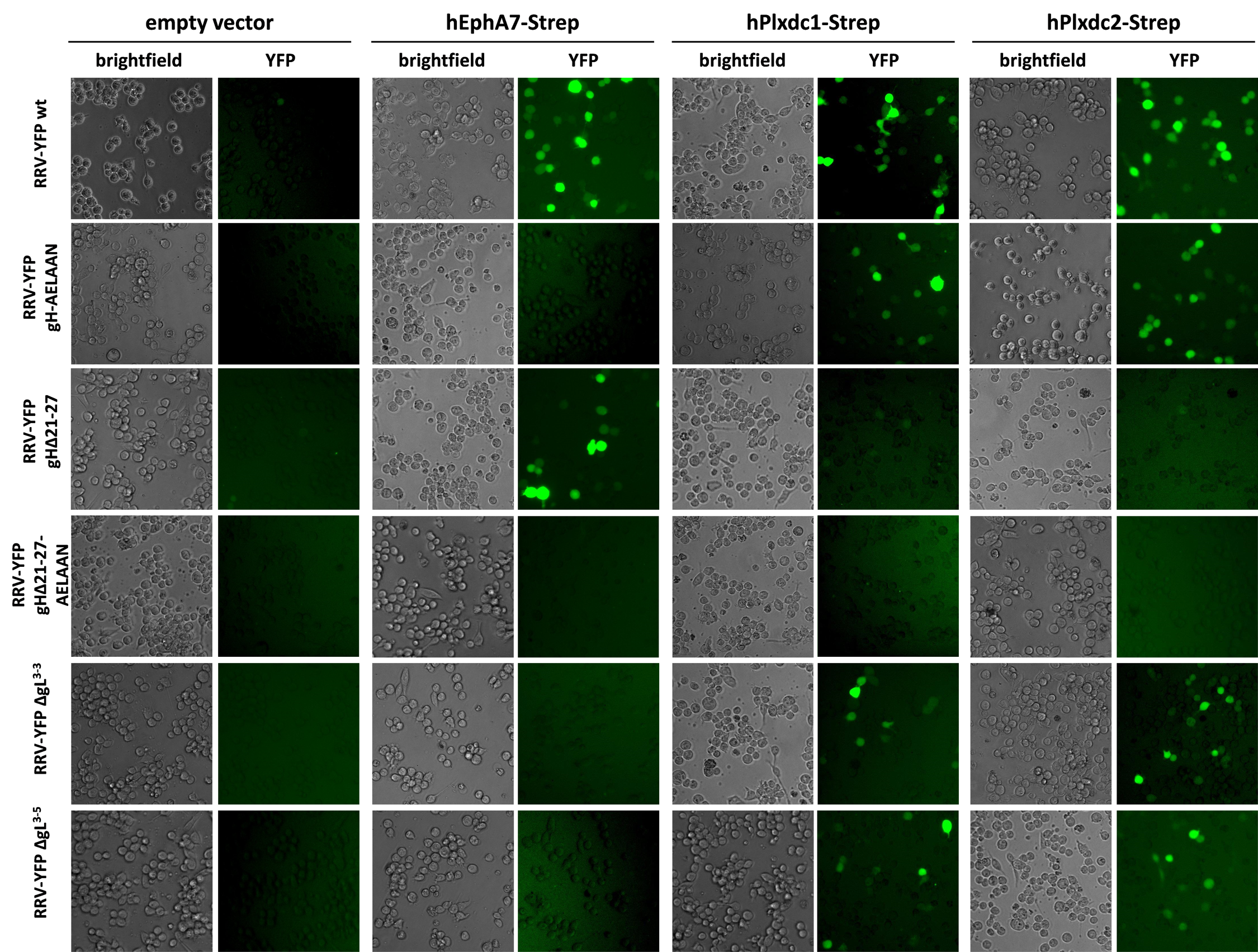

Supplement: S4 Fig — Raji cells were transduced with TwinStrep-tagged human EphA7, Plxdc1 and Plxdc2 (hEphA7-Strep/ hPlxdc1-Strep/ hPlxdc2-Strep) expression constructs or an empty vector control, briefly selected and infected with RRV-YFP wt, RRV-YFP gH-AELAAN, RRV-YFP gHΔ21–27, RRV-YFP gHΔ21-27-AELAAN or one of two RRV-YFP ΔgL clones normalized to genome copies as determined by qPCR. Micrographs show representative infection of the indicated cell pools. (TIF) [file ppat.1008979.s004.tif]
